# Strength training in elderly: An useful tool against sarcopenia

**DOI:** 10.3389/fspor.2022.950949

**Published:** 2022-07-18

**Authors:** Roberto Cannataro, Erika Cione, Diego A. Bonilla, Giuseppe Cerullo, Fabrizio Angelini, Giuseppe D'Antona

**Affiliations:** ^1^Department of Pharmacy, Health and Nutritional Sciences, University of Calabria, Rende, Italy; ^2^Galascreen Laboratories, University of Calabria, Rende, Italy; ^3^Research Division, Dynamical Business and Science Society–DBSS International SAS, Bogotá, Colombia; ^4^Research Group in Physical Activity, Sports and Health Sciences (GICAFS), Universidad de Córdoba, Montería, Colombia; ^5^Sport Genomics Research Group, Department of Genetics, University of the Basque Country (UPV/EHU), Leioa, Spain; ^6^Department of Movement Sciences and Wellbeing, University of Naples Parthenope, Naples, Italy; ^7^JMedical-Sport Medical Center Juventus FC, Turin, Italy; ^8^Centro di Ricerca Interdipartimentale nelle Attività Motorie e Sportive (CRIAMS)-Sport Medicine Centre, University of Pavia, Voghera, Italy; ^9^Department of Public Health, Experimental and Forensic Medicine, University of Pavia, Pavia, Italy

**Keywords:** sarcopenia, strength training, resistance training, muscle hypertrophy, muscle mass loss

## Abstract

The loss of muscle mass and strength in elderly population (especially after the age of 65–70) represents a public health problem. Due to the high prevalence of frailty in older adults, cardiovascular or low-intensity exercise is implemented as first choice option. Although beneficial these training schemes are not as effective as strength-based resistance training for increasing muscle strength and hypertrophy. In fact, when performed progressively and under professional supervision, strength-based training has been proposed as an important and valid methodology to reduce sarcopenia-related problems. In this mini-review, we not only summarize the benefits of weight resistance training but also highlight practical recommendations and other non-conventional methods (e.g., suspension training) as part of an integral anti-sarcopenia strategy. Future directions including cluster set configurations and high-speed resistance training are also outlined.

## Introduction

The loss of muscle strength (dynapenia) is one of the most evident and problematic conditions in older population (Neves et al., 2018). This phenomenon is present in both women and men with higher prevalence in the first, and important consequences in the quality of life (QOL) besides predisposition to injuries and pathologies such as type II diabetes and sarcopenia (Cannataro et al., 2021a). After more than 30 years of clinical research, the emphasis has been placed on increasing physical activity in elderly population, although the importance of physical exercise has raised recently (Broeder et al., 1992). This mini-review aims to highlight the superiority of progressive and supervised strength-based training compared to other methods. This will be covered not only in health but also in disease phenotypes given heavy-load strength training may reduce illness symptoms while improving physical condition and QOL (Malorgio et al., 2021).

Aging is associated with a loss in both muscle mass and in the metabolic functionality of skeletal muscle, a phenotype designed as sarcopenia by Rosenberg (1997). Muscle mass in subjects between the ages of 50 and 70 decreases by about 8% per year (Lee et al., 2018). Although the concept of sarcopenia is well established, there is still no unanimous consensus for the characterization and therefore the diagnosis. At present diagnosis of sarcopenia come from both loss of muscle strength and performance; in clinical practice, the tools utilized are a handheld dynamometer to assess strength handgrip and the Short Physical Performance Battery or simply gait speed to assess physical performance; subsequently, other exams could be performed to directly analyze muscle mass and fat infiltration, Dual-energy X-ray Absorptiometry (DXA) and Bioelectrical Impedance Analysis (BIA) are performed, but the gold standard is Magnetic Resonance Imaging (MRI) or Computed Tomography (CT); finally ultrasound technique is increasing in use, but at the moment not in a standardized manner (Liguori et al., 2018). Consequently, the countermeasures are of various types and not uniform. This condition mainly results from adaptive changes after a decrease in the physical activity level and may subsequently be counteracted by improving physical fitness (Fielding et al., 2017); however, senescence and genetic-related individual responses should not be omitted (Singh and Gasman, 2020). Thus, risk factors that determine this condition are multifactorial in nature and include genetic factors, sex, age, medical history, comorbidities, social factors and physical activity level (Supriya et al., 2021). This condition is aggravated if at the same time there is overweight or obesity. As discussed in our recent review (Cannataro et al., 2021a), nutrition and dietary supplementation have a relevant role as physical exercise within the integral multidisciplinary approach to combat sarcopenia. It is worth noting that Fritzen et al. (2020) showed how the ability to respond to strength exercise is comparable in young and older adults, with the deconditioning followed a similar time course. The general recommendations regarding physical activity are addressed to prevent mobility decline and involve a combination of resistance training and cardiovascular activities (Cruz-Jentoft et al., 2019; Fritzen et al., 2020; Singh and Gasman, 2020; Supriya et al., 2021). In the case of resistance training, the American College of Sport Science (ACSM) (Chodzko-Zajko et al., 2009) has suggested a training frequency ranging between 2 and 4 days weekly and a low training volume [60–80% of one-repetition maximum (1RM)] for major muscle groups, that it could be a good starting point but it should be “personalized” and if possible increased in intensity, volume and frequency. Therefore, the loss of muscle mass with advancing age can be considered as an almost physiological process but it can be certainly slowed down by performing strength-based resistance exercises.

## Strength training

Strength training is a type of physical exercise that is performed by contracting skeletal muscle fibers to generate work against a given weight or external force. While strength training is more specific, resistance training may use several forms of external resistances such as body mass, weights or resistance bands to improve muscle strength, power, hypertrophy and endurance (Stone et al., 2002). The musculoskeletal tissue possesses remarkable plasticity in response to repeated stimuli, such as resistance training. If the biological stress is sustained for an appropriate interval of time by allowing optimal recovery (between both sets and exercise sessions) the desired phenotypic changes might occur (e.g., increase in the ability to sustain contraction, increase strength, etc.) (Bonilla et al., 2020). In effect, the most important principles of exercise training should be followed:

- An overload should be applied: the positive adaptation occurs only if the actual training load overcome the habitual level.
- Specificity and individualization: all exercisers are different based on training experience and genetics (even in the same age range).
- Periodization: training load (e.g., intensity, volume) must vary over the time to avoid accommodation.

All principles converge to establish optimal allostatic load (Guidi et al., 2021) and/or supercompensation (reach a level above the initial value) (Impellizzeri et al., 2019). For this reason, it is necessary a careful and qualified supervision particularly during sarcopenia conditions in aging subjects. This might guarantee an adequate progression of the training schemes and their execution. We have, in fact, shown in two case reports how two women aged 52 and 72 with osteoarticular pathologies (spondyloarthritis and osteoarthritis, respectively) have obtained significant improvements in powerlifting performance (+100 kg in deadlift) and in QOL (Cannataro et al., 2021b; Malorgio et al., 2021). From the basic strength conditioning to specific results, they spend 2 and 5 years, respectively. It is worth mentioning the ACSM (2019) highlights regarding the benefits after following a strength-based resistance training program–best if the program includes a cardiovascular training component (Bonilla et al., 2021):

- Muscle strength, endurance and power.
- Bone mineral density and connective tissue remodeling.
- Cardiometabolic health through tissues cross-talking.
- Growth hormone and blood glucose regulation.
- Less frailty, avoid falls and improved functionality.

Even though cardiovascular exercise (low-intensity or intermittent training) has been shown to improve insulin sensitivity and glucose tolerance *via* glucose transporter 4 (GLUT4) regulation, robust evidence highlight how resistance training also offers advantages in glycemic regulation (Impellizzeri et al., 2019; Guidi et al., 2021). Last but not least, strength training has also anti-inflammatory actions due to its exclusive effect on mechano growth factor (MGF) (Guidi et al., 2021) and downregulation of the tumor necrosis factor α (TNFα) pathway (Impellizzeri et al., 2019). This might be linked to the improved action of insulin and blood glucose regulation (Dela and Kjaer, 2006).

## Non-traditional resistance training

Various types of resistance training have been proposed as useful approaches for improving body composition, muscle strength, power, and physical function in older adults (Baena-Marín et al., 2022). Among these, suspension-based resistance training (S-RT) involves attaching body segments to suspended hanging straps, creating an unstable environment, and performing multi-planar and multi-joint exercises while fighting gravity (Byrne et al., 2014; Mok et al., 2015; Cugliari and Boccia, 2017; Consitt et al., 2019; Cannataro et al., 2021b). Research on the acute effects of S-RT has shown similar results compared to traditional weight resistance training conducted in a stable environment (Sun et al., 2018; Jiménez-García et al., 2019). For example, a 12-weeks program of S-RT promotes similar improvements on muscle mass, strength and physical performance compared to traditional resistance training in older adults (Soligon et al., 2020; Campa et al., 2021). Furthermore, the benefits on performance after S-RT might involve greater activation of core muscles. In this sense, results from a recent systematic review showed higher activation of core muscles for S-RT compared to weight resistance training in the push-up, inverted row, prone bridge, and hamstring curl exercises (Aguilera-Castells et al., 2020). Other studies have also shown that S-RT might be an efficient strategy to improve body composition, sleep quality and fatigue tolerance in both men and women (Campa et al., 2018, 2021; Chen et al., 2018; Vikberg et al., 2019; Jiménez-García et al., 2021; Hayes et al., 2022). As expected, a program of 8-weeks of S-RT has shown benefits on glycemic and lipid profiles in women with Type 2 Diabetes (Samadpour Masouleh et al., 2021). Since it provides positive effects in muscle mass and substantial activation of core muscles, the combination of strength, mobility and balance training by means of a S-RT program might be a promising strategy to improve body composition (increase fat-free mass) while increasing muscle strength, power, and functional performance in older adults, as suggested recently by Angleri et al. (2020). It needs to be noted that further studies are warranted to have practical recommendations of S-RT in the elderly population.

On the other hand, advanced training methodologies have been studied to accelerate exercise-induced adaptations in several populations. For example, cluster set configurations have been implemented in resistance training to optimize sports performance (Tufano et al., 2017). Although cluster-set resistance training protocols are more oriented to increase performance and strength levels, they may also be a useful tool to increase total training volume which has been found as the main training stimuli behind muscle hypertrophy (Vargas-Molina et al., 2021). A cluster set resistance training method is applied in 3–5 RM blocks, with a total of 3–4 blocks, and intra-set rest periods are between 20 and 30 s. We have previously demonstrated that four blocks of 3RM with 20 s of intra-set pause were more effective for increasing fat-free mass evaluated by DXA compared to four blocks of 3RM with 40 s of intra-set pause or two blocks of 6RM with 20 s of intra-set pause in healthy resistance-trained men (Vargas-Molina et al., 2020). Interestingly, the cluster set configuration has shown to be equally effective to muscle strength/power and functionality compared to traditional resistance training in post-menopausal and elderly women (Dias et al., 2020). Notwithstanding, high-speed resistance training using a cluster set configuration has resulted in significantly greater improvements in functional performance and QOL in elderly women (66.5 ± 5.4 years) in comparison to the traditional resistance training methodology (Ramirez-Campillo et al., 2018). It is worth mentioning that velocity-based resistance training is a methodology based on monitoring and dosing the training load by measuring movement velocity (Baena-Marín et al., 2022). In this regard, a recent study by Vieira et al. (2022) showed that 8 weeks of high-speed resistance training was able to promote improvements on functionality with greater effects on strength gains compared to traditional methods in older adults (67.8 ± 6.3 years). There is no doubt that next studies will help to corroborate these findings and have a clearer panorama to recommend cluster sets and high-speed resistance training in elderly population.

## From bench (lab) to bench press: putting into practice

The objective of this work is not to determine which type of physical exercise is best to apply in adults aged +50 years but emphasizes on the effectiveness of strength-based resistance training even with high loads during a well-organized periodized and monitored program (Vargas-Molina et al., 2022). As above mentioned, there are no substantial differences in the physiological response to strength training between young and older adults; however, it is necessary to consider existing and potential osteoarticular- and cardiovascular-related pathologies. For this reason, careful supervision by qualified personnel with sufficient experience is essential. In this sense, Mañas et al. (2021) reviewed a series of studies that evaluated at-home self-administered and self-monitored resistance training using elastic bands, free weights or body mass with negligent results despite the good compliance which highlight the need for progressive overload. To reinforce the importance of adequate supervision, Vikberg et al. (2019) showed that exercise professional supervision during a low-load training program produced significant improvements in 70-year-old participants. Similar positive results were obtained by Björkgren et al. (2021). Chen et al. (2018) demonstrated the effectiveness and safety of a medium-to-high load kettlebells-based resistance training program that included multi-joint exercises (i.e., deadlift and squat) in participants between 65 and 75 years old. Interestingly, the results were maintained for 4 weeks after the duration of the study (8 weeks). The same research group previously reported positive variations on IGF-1 concentrations in older adults with sarcopenic obesity which may be explain at least in part the improvements due to strength training (Chen et al., 2017). It needs to be noted that exercise selection might highly impact the exercise response in the elderly; for example, multi-joint exercises such as squats or deadlifts provide a greater stimulus to hypertrophy by involving larger muscle groups and require the use of core stabilizing muscles which has been shown to maintain good posture (Stone et al., 2002).

**Figure 1:**
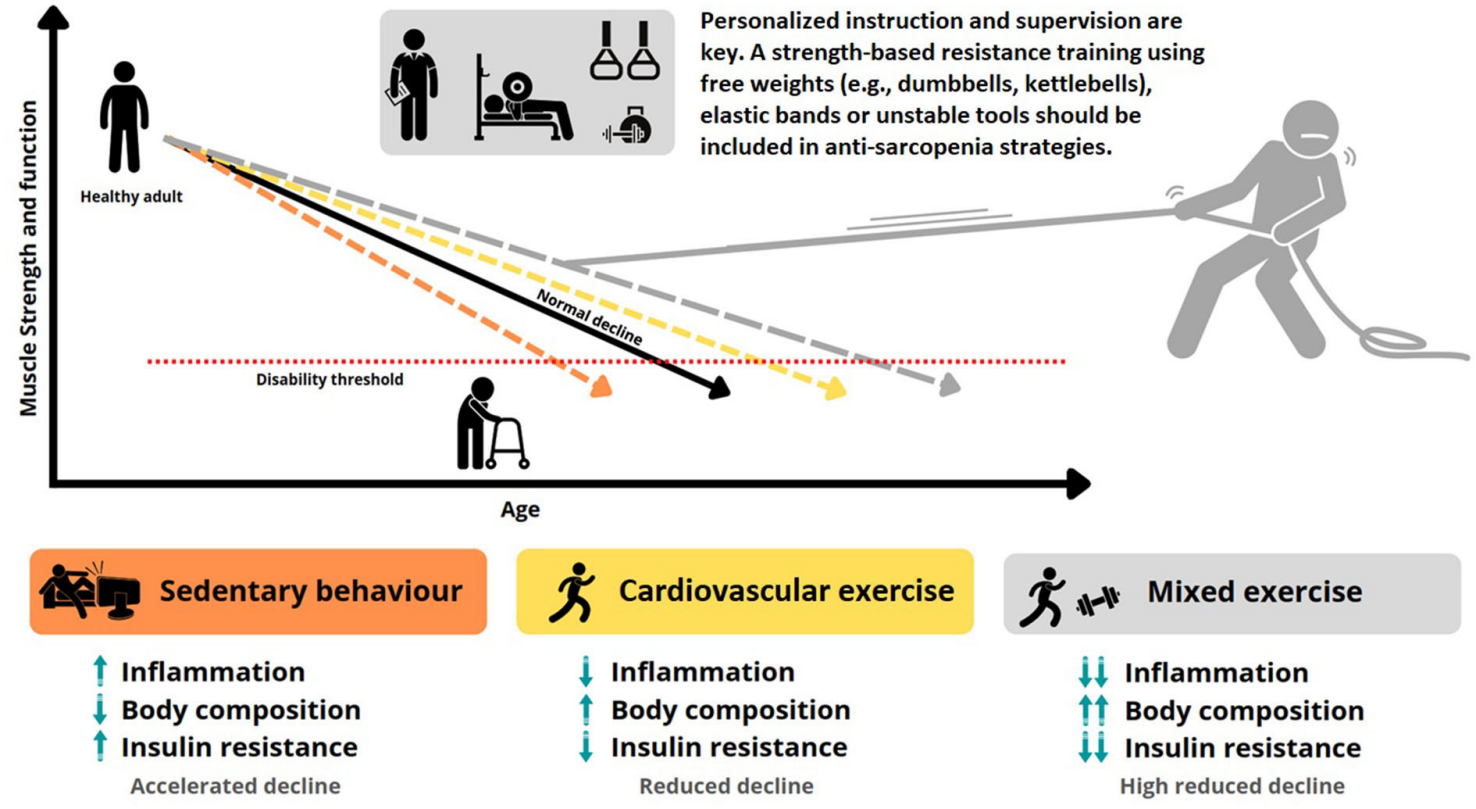
Training approaches to sarcopenia.

By scoping the literature, Hayes et al. (2022) raised a relevant aging-related phenomenon, the time course of the adaptive response to the physical exercise. Although exercise-induced adaptations can be comparable, the time to reach the new set point and acquire exercise adaptations is certainly greater in older subjects compared to young people. The literature is still limited and, thereby, an accurate mechanism or practical recommendations cannot be given; nevertheless, adaptations will hypothetically take more time in elderly population than in young subjects due to biological senescence. This represents a critical call to action related the assessment of efficacy/safety ratio by monitoring training load (intensity and volume) since older adults might respond differently and require longer recovery times (Ribeiro et al., 2020). For instance, older adults not accustomed to exercise might misclassify perceived exertion (potential discomfort) (Jabbour and Majed, 2018) besides finding physical training as time-consuming which increase the probability of abandonment (Chao et al., 2000). In weight resistance training, the intensity is generally measured as a percentage of 1RM (i.e., the maximum load with which you can perform a single repetition) although this is not suitable for older individuals due to the high stress generated by the 1 RM test. Hence, it has been suggested to estimate it based on 6 or 10 RM (Cruz-Jentoft et al., 2019; Fritzen et al., 2020). From a practical point of view, the OMNI-RES perception scale has been used in older adults of both sexes to help gain insight about perceptions of effort during exercise (Gearhart et al., 2009). The rating of perceived exertion scales has been recognized as a valid marker of internal load (Falk Neto et al., 2020). This easy-to-apply methodology help to accurately monitor intensity taking advantage of the interoception process as control within the allostasis model (Bonilla et al., 2020). An alternative strategy can be implementing low-load power-based training which applies a faster movement execution with less recovery time between sets, especially in conditions of advanced sarcopenia or other concomitant pathologies (Sun et al., 2018; Mañas et al., 2021). Health, exercise and strength conditioning professionals may use the scale of perception of movement velocity in resistance exercise that was developed and validated by Bautista et al. (2014). All the concepts described above are schematized in Figure 1.

## Conclusions

It is clear that strength-based resistance training with or without other anti-sarcopenia strategies (e.g., high-protein diet, creatine supplementation, etc.) represent a safe and effective methodology to avoid the progress of sarcopenia by improving changes in body composition (increase muscle mass) and functionality that are affected by the aging process. Individualized and periodized resistance training programs might act against sarcopenia progression/development by improving muscle strength, providing a hypertrophic stimulus that may contribute to the maintenance of muscle mass, improving immunological surveillance, and favoring correct posture. Nevertheless, some weaknesses need to be underlined including the requirement of qualified supervision, the learning path of exercise movement (technique), and the less tolerance to pain in not accustomed individuals due to delayed onset of muscle soreness. General population and health/exercise professionals should be aware that the combination of strength and endurance training might probably be an optimal strategy to improve adherence. Finally, utilizing the OMNI-RES and the scale of perceived movement velocity might provide important information to monitor training load in order to optimize strength training-induced adaptations in older adults.

## Author contributions

Conceptualization: RC, EC, and DAB. Writing—original draft preparation: RC and EC. Writing—review and editing: DAB, GC, FA, and GD'A. All authors have read and agreed to the published version of the manuscript.

## Conflict of interest

DAB has conducted academic-sponsored research on resistance training, and has received honoraria for selling linear position transducers and speaking about velocity-based resistance training at international conferences/private courses. All authors are responsible for the content of this article. The remaining authors declare that the research was conducted in the absence of any commercial or financial relationships that could be construed as a potential conflict of interest.

## Publisher's note

All claims expressed in this article are solely those of the authors and do not necessarily represent those of their affiliated organizations, or those of the publisher, the editors and the reviewers. Any product that may be evaluated in this article, or claim that may be made by its manufacturer, is not guaranteed or endorsed by the publisher.
